# Coding, modifier, and regulatory effects shape circulating APOL1 levels

**DOI:** 10.1093/hmg/ddag087

**Published:** 2026-09-18

**Authors:** Walt E Adamson, Harry Noyes, John Ogunsola, Rulan S Parekh, Anneli Cooper, Annette MacLeod

**Affiliations:** School of Biodiversity, One Health, and Veterinary Medicine, University of Glasgow, Glasgow, G12 8QQ, UK; Centre for Genomic Research, University of Liverpool, Crown Street, Liverpool, L69 7ZB; School of Biodiversity, One Health, and Veterinary Medicine, University of Glasgow, Glasgow, G12 8QQ, UK; Women’s College Hospital, Hospital for Sick Children and University of Toronto, Toronto, M5S 1B2, Canada; School of Biodiversity, One Health, and Veterinary Medicine, University of Glasgow, Glasgow, G12 8QQ, UK; School of Biodiversity, One Health, and Veterinary Medicine, University of Glasgow, Glasgow, G12 8QQ, UK

**Keywords:** genetic modifiers, population genetics, regulatory genomics, human genetics

## Abstract

*APOL1* variants G1 and G2 are major genetic risk factors for chronic kidney disease in people with recent sub-Saharan African ancestry, yet the mechanisms linking genotype to disease remain poorly defined. Circulating APOL1 is likely to play a critical role, although how specific genotypes and regulatory variants influence circulating APOL1 levels is incompletely understood. We analysed Olink-measured APOL1 NPX (Normalized Protein eXpression) levels from 43 587 UK Biobank participants. Associations between APOL1 NPX and known genotypes were assessed in individuals with recent sub-Saharan African ancestry, including analyses of G1, G2, E150K (rs2239785) and N264K (rs73885316). We also investigated protein quantitative trait loci and cis-acting variants across the *APOL1* locus, including predicted microRNA binding sites. Results indicated that APOL1 NPX varied in an *APOL1* genotype-dependent manner. E150K, G1, and G2 demonstrate a clear dose-dependent relationship with APOL1 NPX, with the largest effect for G2. After accounting for *APOL1* genotype, APOL1 NPX remained higher in individuals with self-reported Black or Black British ethnicity, consistent with additional regulatory elements. Multiple cis-regulatory variants near *APOL1* were independently associated with APOL1 NPX, and are likely to contribute to ancestry-related differences. Together these findings demonstrate that *APOL1* genotype is a major determinant of APOL1 NPX, and that G1 and G2 have distinct biological effects. Regulatory variation adds another layer of control over APOL1 NPX and may help explain population differences. These findings provide a biological framework for understanding genotype-specific molecular effects of *APOL1* variation.

## Introduction

Chronic kidney disease (CKD) disproportionately affects individuals of recent sub-Saharan African ancestry [1]. This excess risk is partly attributable to two independent missense variants of the apolipoprotein L1 (*APOL1*) gene: G1 (rs73885319 and rs60910145) and G2 (rs71785313) [2], which are common in sub-Saharan Africa and its diaspora, but rare in other populations. G1 (amino acid substitutions S342G and I384M) and G2 (deletion of N388 and Y389) are found in the same domain at the C-terminus of *APOL1* and exhibit complete linkage disequilibrium: haplotypes with both G1 and G2 alleles are either very rare or absent. As a result, six combined genotypes with respect to *APOL1* G1 and G2 have been observed (Table 1).

**Table 1:** The six observed combined genotypes with respect to the G1 and G2 loci. Genotypes with G1 and G2 on the same haplotype are theoretically possible but have not been observed.

Genotype name	Haplotypes		Variant copy number	G1 copy number	G2 copy number
	G1 locus	G2 locus			
G0/ G0	AT AT	TTATAA TTATAA	0	0	0
G0/ G1	AT GG	TTATAA TTATAA	1	1	0
G0/ G2	AT AT	TTATAA 6 bp deletion	1	0	1
G1/ G1	GG GG	TTATAA TTATAA	2	2	0
G1/ G2	GG AT	TTATAA 6 bp deletion	2	1	1
G2/ G2	AT AT	6 bp deletion 6 bp deletion	2	0	2

APOL1 also plays a crucial role in protecting humans from the highly pathogenic trypanosome parasite, *Trypanosoma brucei* by forming channels in the parasite’s membrane, leading to osmotic imbalance and cell death. However, two subspecies of *Trypanosoma brucei, T. b. gambiense* and *T. b. rhodesiense*, have evolved mechanisms to resist APOL1’s action and so can cause the often-fatal disease, human African trypanosomiasis. The G1 and G2 variants of *APOL1* are associated with protection from human African trypanosomiasis [3]. In *T.b. gambiense* infections, G1 and G2 are associated with decreased and increased risk of severe disease, respectively, while carriage of the G2 variant protects against infection by *T.b. rhodesiense* [3]. Despite the association of different genotypes with distinct phenotypes in human African trypanosomiasis, studies of the association between kidney diseases and *APOL1* have often grouped the G1 and G2 variants together as recessively ‘high-risk’ in any combination. Carriage of two such alleles (i.e. G1 homozygotes, G2 homozygotes, and G1/G2 compound heterozygotes) is associated with a spectrum of kidney and related conditions including focal segmental glomerulosclerosis, hypertension-associated kidney failure, and HIV-associated nephropathy [2, 4–6].

Association studies examining *APOL1* variants are often limited in statistical power and have not reported data consistently by haplotype. In a prior study using UK Biobank data, we previously highlighted differences in association between the three two-variant *APOL1* genotypes (G1/G1, G1/G2, and G2/G2) and kidney phenotypes, and demonstrated that the compound heterozygous genotype, G1/G2 (carried by millions of people worldwide), is deleteriously associated with 26 different conditions spanning human health [7]. In contrast, G1/G1 and G2/G2 genotypes were only associated with kidney conditions. Our analysis exposed complexities in the relationship between *APOL1* risk alleles and disease that are not evident when two-variant *APOL1* genotypes (G1/G1, G1/G2, and G2/G2) are grouped together as a homogeneous risk category.

*APOL1* also contains additional variants that may modify its effects on kidney disease and trypanosome infections. One such variant, N264K, was initially identified in an individual with the G2/G2 genotype, who was infected with an atypical *T.b. gambiense* strain. Parallel *in vitro* experiments demonstrated a reduction in the ability of APOL1 to lyse trypanosomes if N264K was present [8]. This reduced trypanolytic activity was mirrored by a reduction in cytotoxicity in kidney cells (HEK293) as a result of APOL1 expression when N264K was present alongside G1 and G2 [9]. Subsequently, it was shown that N264K, when co-inherited with G2, also reduces the renal toxicity associated with the G1/G2 and G2/G2 genotypes [10, 11].

Given the recessive inheritance and incomplete penetrance, it is not clear how APOL1 causes cell injury in CKD. It is likely that there are both genetic and environmental factors are likely to contribute to disease development. A minor fraction of APOL1 protein is expressed by the kidney and other tissues but the majority is produced by the liver and circulates bound in protein complexes as a component of plasma HDL. The relative contributions of endogenous APOL1 production in the tissues and of circulating APOL1 from the liver to disease processes remain a point of investigation. Serum APOL1 levels have been correlated with some pathologies such as sepsis and COVID-19 severity [12], whereas other studies found no correlation with kidney disease risk [13].

Recent work has shown that how APOL1 plasma concentrations are measured (affinity-based assays and mass spectrometry) can be influenced by APOL1 coding variants, with discordant directions across affinity-based assays and no significant effect in mass spectrometry [14]. G1, G2, and also rs2239785 (E150K) missense coding variants were shown to have inconsistent associations with APOL1 concentration measured by different platforms. These discrepancies appear to arise from protein-altering variants that might influence epitope recognition or conformational state. This suggests that affinity-based assays may detect conformational or epitope-dependent properties of APOL1 rather than absolute circulating abundance. These observations raise important questions about how circulating APOL1 measured by affinity assays should be interpreted in genetic studies. Even if affinity-based measurements do not directly reflect total protein concentration, they may capture biologically meaningful genotype-dependent differences in circulating APOL1 isoforms, structural states, or protein complexes. Characterising the genetic architecture underlying this plasma APOL1 signal, including coding and non-coding regulatory effects, remains essential for understanding how APOL1 genotype influences systemic biology and potentially contributes to disease risk.

We analysed circulating APOL1 Normalized Protein eXpression (NPX) using Olink proteomics in UK Biobank participants [15, 16], aiming to characterise the genetic and regulatory architecture underlying APOL1 NPX. Previously a genome-wide association study screening for associations with protein concentration in the UK Biobank identified *APOL1* G2 (alongside SNPs rs138477541 or rs1053865678 on chromosomes 3 and 5 respectively) as having an association with APOL1 concentration among people who self-reported Black/Black British [16] and rs2239785 (E150K) and rs6000220 were also associated with APOL1 NPX in a subsequent study [14]. Here, we examined differences in circulating APOL1 NPX across ethnic groups. We then examined the impact of *APOL1* regulatory variants on APOL1 NPX: first by scanning for associations between APOL1 NPX and SNPs in the 1 MB region around *APOL1*, and then testing whether the resulting cis-pQTLs were within microRNA binding sites that might regulate APOL1 NPX. By integrating genotype-specific effects, population differences, and cis-regulatory architecture, we define the structured genetic architecture underlying circulating APOL1 signal.

## Results

### APOL1 NPX varies by self-reported ethnicity

Among the 53 018 UK Biobank participants for whom Olink proteomic data was available, the mean age at recruitment and blood sampling was 56.8 years, 54% were female, while 93.3% and 2.3% self-reported White and Black ethnicity respectively. Olink data are expressed in the Normalized Protein eXpression (NPX) scale, enabling relative quantification of the same protein across multiple batches. As NPX values are calculated on a log2 scale, an NPX difference of 1 represents a doubling of protein concentration.

There were 43 587 participants with APOL1 NPX, valid combinations of G1 and G2 genotypes, and self-defined ancestry within the five major ethnic groups (Table 2). A linear model with sex, age, and the first four genetic principal components was used to test for associations with age and sex. Female participants had significantly higher APOL1 NPX (0.16) than males (−0.02) (*P* = 3.4 × 10–163; β = 0.16 ± 0.01; OR = 0.85). APOL1 NPX declined significantly with age from 0.23 at 40 years to −0.11 at 70 (*P* = 4.5 × 10^−94^; β = −0.0074 ± 0.0007; OR 0.99).

**Table 2:** APOL1 NPX values by ethnicity and genotype. NPX values are calculated on a log2 scale. The number in each group is the number of participants with complete and valid data for APOL1 NPX, self-reported ethnicity, G1/G2 haplotype and rs2239785 genotype. rs2239785. Therefore any variation in APOL1 NPX between participants homozygous for the reference alleles at these loci should be attributable to differences in regulation and not gene structure.

	All Genotypes			Homozygous reference G1, G2, rs2239785 (E150K)		
Self-reported ethnicity	N	Mean APOL1 NPX	95% CI APOL1 NPX	N	Mean APOL1 NPX	95% CI APOL1 NPX
Asian/Asian British	843	−0.149	(−0.184–0.115)	587	−0.174	(−0.214–0.135)
Black/Black British	1000	1.101	(1.049–1.153)	121	0.349	(0.236–0.462)
Chinese	135	−0.107	(−0.186–0.028)	91	−0.113	(−0.216–0.009)
Mixed	283	0.383	(0.287–0.478)	139	0.143	(0.025–0.261)
White	41 326	0.067	(0.061–0.073)	27 146	0.004	(−0.003–0.011)

We compared APOL1 NPX across self-reported ethnicities as defined by the UK Biobank: Asian or Asian British (which includes Indian, Pakistani, and Bangladeshi ethnicities); Black or Black British; Chinese; Mixed; White. All comparisons between self-reported ethnicities yielded significant differences (*P* < 3x10^−5^), except for the comparison between Asian and Chinese ethnicities (*P* = 0.74) (Table 3, Fig. 1). UK Biobank participants self-reporting Black or Black British ancestry had almost two-fold higher NPX of APOL1 than other ethnicities.

**Table 3:** Pairwise comparisons of mean APOL1 NPX between self-reported ethnicities using age- and sex-adjusted linear regression models. Results are shown for all participants and for the subset homozygous for the reference alleles at G1, G2, and rs2239785 (E150K). Beta values represent the differences in APOL1 NPX relative to the comparison group.

Ethnicity 1	Ethnicity 2	All participants				Reference genotype at G1, G2 and rs2239785 (E150K) loci			
		Beta	Beta 95% CI	OR	*P*	Beta	Beta 95% CI	OR	*P*
Asian	Black	1.218	1.15, 1.28	3.38	6 × 10^−221^	0.47	0.37, 0.57	1.60	7 × 10^−20^
Asian	Chinese	0.015	−0.08, 0.11	1.02	0.74	0.02	−0.08, 0.13	1.02	0.67
Black	Chinese	−1.211	−1.36, −1.07	0.30	9 × 10^−55^	−0.46	−0.61, −0.3	0.63	2 × 10^−08^
Mixed	Asian	−0.49	−0.57, −0.41	0.61	5 × 10^−30^	−0.25	−0.35, −0.15	0.78	7 × 10^−07^
Mixed	Black	0.741	0.63, 0.85	2.10	4 × 10^−37^	0.22	0.06, 0.39	1.25	0.007
Mixed	Chinese	−0.483	−0.63, −0.33	0.62	5 × 10^−10^	−0.24	−0.40, −0.07	0.79	0.006
White	Asian	−0.23	−0.27, −0.19	0.79	3 × 10^−27^	−0.19	−0.24, −0.14	0.83	2 × 10^−14^
White	Black	0.99	0.95, 1.03	2.69	< 10^−221^	0.30	0.20, 0.41	1.35	2 × 10^−08^
White	Chinese	−0.219	−0.32, −0.12	0.80	3 × 10^−05^	−0.16	−0.28, −0.04	0.85	0.01
White	Mixed	0.251	0.18, 0.32	1.29	7 × 10^−12^	0.08	−0.02, 0.18	1.08	0.12

**Figure 1:**
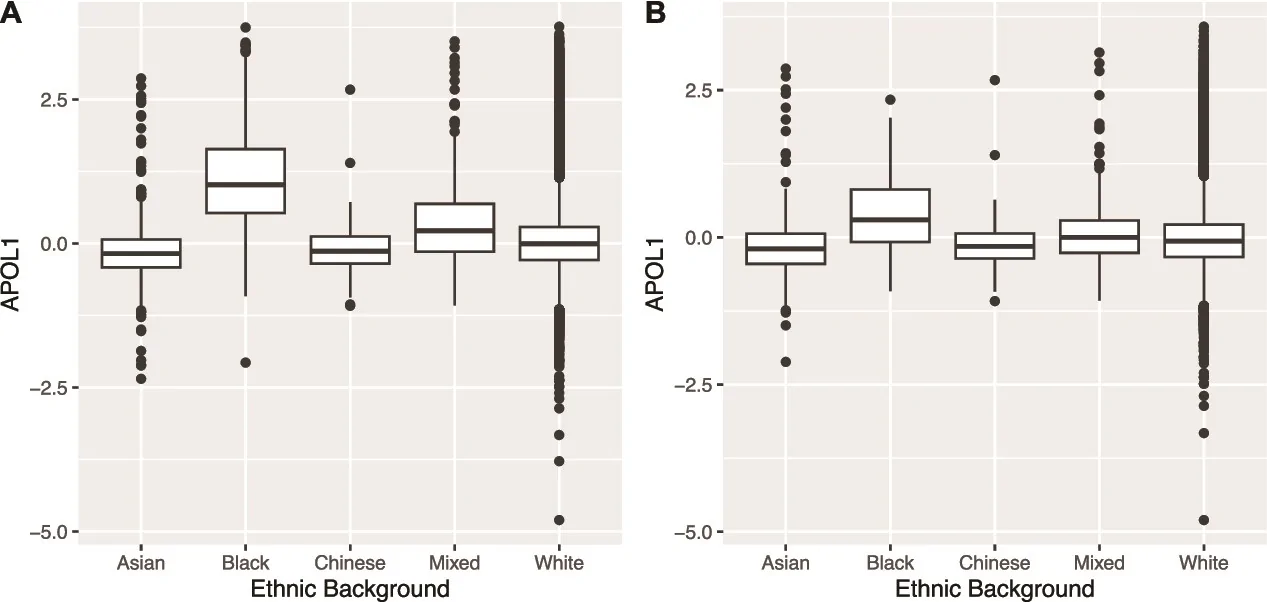
Distribution of APOL1 NPX for each self-reported ethnicity (as defined by the UK biobank). (A) Using data from all participants; (B) using data from participants homozygous for the reference non-synonymous alleles at G1, G2 and E150K. Some of the differences between ethnicities in A are attributable to non-synonymous SNPs causing changes in antibody binding whilst differences in B are assumed to reflect differences in APOL1 NPX levels associated with regulatory differences.

To begin exploring the genetic factors contributing to this observation, we investigated the impact of *APOL1* genotypes on circulating APOL1 NPX levels. Olink APOL1 NPX data and complete *APOL1* genotypes were available for 1000 UK Biobank participants with self-reported Black or Black British ethnicity, among whom the mean age was 51.4 years and 54% were female.

### APOL1 NPX increases with number of G1 or G2 alleles

Our previous phenome-wide analysis highlighted differences in associations with health conditions between the six observed combined *APOL1* G1 and G2 genotypes [7], with two-variant *APOL1* genotypes being associated with distinct kidney phenotypes, and the G1/G2 genotypes being uniquely associated with a range of conditions spanning human health. To investigate this further we used a linear model with age, sex, and the first four genetic principal components as covariates to examine the association of APOL1 NPX levels with each *APOL1* genotype among UK Biobank participants who self-report Black or Black British ethnicity. APOL1 NPX increases with the number of G1 and G2 alleles carried (Table 4). G2 homozygotes (G2/G2) had exceptionally high APOL1 NPX, all carriers of this genotype were in the top 2.6% of the APOL1 NPX distribution for the whole UK Biobank populations and in the top 16% of the population with sub-Saharan African ancestry. Linear regression confirmed the allele count effects: G1 alleles were associated with modest increases (β 0.32; 95% CI (0.27, 0.38); OR 1.4; *P* = 8 × 10^−27^), while G2 alleles had stronger effects (β = 1.26; 95% CI 1.18–1.34; OR 3.51; *P* = 3 × 10^−149^). Interestingly, individuals with the G0/G2 genotype, which is not associated with an increased risk of CKD, had higher APOL1 NPX levels than those with the G1/G1 genotype (β = 0.73, 95% CI (0.61, 0.86), OR = 2.1, *P* = 2 × 10^−23^), who are at increased CKD risk. Thus, disease risk associated with G1 and G2 alleles cannot be explained solely by plasma APOL1 NPX. The G1/G2 genotype appeared additive, with NPX levels reflecting the combined effects of G1 and G2, suggesting that its broad phenotypic impact is not associated with APOL1 NPX. Notably, the distinct NPX distributions observed across G1/G1, G1/G2, and G2/G2 genotypes indicate that the *APOL1* variants produce measurably different signals, consistent with underlying structural or biochemical differences between the variants. Carriage of *APOL1* G1 and G2 variants is extremely rare in populations that do not have recent sub-Saharan African ancestry; therefore it was not possible to assess the impact of G1 and G2 in other self-reported ethnicities.

**Table 4:** Mean APOL1 NPX and genotype effects associated with each APOL1 genotype in black/black British participants. Beta coefficients were estimated using age- sex-, and genetic principal component-adjusted linear regression models. Effect estimates are shown relative to the G0/G0 reference genotype.

APOL1 genotype	Participants (n)	Mean APOL1 NPX	OR	Beta	Beta 95% CI	*P*
G0/G0	448	0.60	-	-	-	-
G0/G1	286	1.05	1.42	0.35	0.26, 0.43	1.7 × 10^−15^
G0/G2	136	2.12	4.07	1.40	1.29, 1.51	2.9 × 10^−111^
G1/G1	78	1.40	1.94	0.66	0.52, 0.79	4.2 × 10^−21^
G1/G2	31	2.37	5.12	1.63	1.43, 1.83	6.7 × 10^−51^
G2/G2	21	2.89	8.28	2.11	1.87, 2.35	7.9 × 10^−58^

### APOL1 rs2239785 (E150K) only modifies APOL1 NPX on a G0/G0 background

rs2239785 (E150K) is the only other common non-synonymous APOL1 variant previously associated with circulating APOL1 NPX. We therefore examined allele frequencies among White British and Black British participants. The frequency of the ancestral E150K G allele differed markedly between White British and Black British participants (34% versus 64%, respectively), although rs2239785 was in Hardy–Weinberg equilibrium in both populations (*P* > 0.7). In Black British participants, the G allele was present on G0, G1 and G2 haplotypes whereas the A allele occurred almost exclusively on G0 chromosomes (Table S1). Consequently, individuals carrying only variant APOL1 haplotypes (G1/G1, G2/G2 or G1/G2), who comprised 13% of the cohort, accounted for just 2.6% of all A alleles. Despite this striking haplotype distribution, rs2239785 showed little linkage disequilibrium with either G1 or G2 (R^2^ < 0.05).

E150K was strongly associated with increased APOL1 NPX in individuals with a G0/G0 genotype (β = 0.261; 95% CI (0.18, 0.34); *P* = 2.5 × 10^−9^). In contrast, E150K had no significant effect on APOL1 NPX when carried on G1 or G2 haplotype backgrounds (β = 0.113; 95% CI (−0.02, −0.24), *P* = 0.089), most likely because the A allele is extremely uncommon on these chromosomes (Table S1). These findings indicate that the influence of E150K on circulating APOL1 NPX is largely restricted to individuals carrying two G0 alleles.

### APOL1 NPX levels in the G0/G0 genotype vary by self-reported ethnicity

Among UK Biobank Black/Black British participants, mean APOL1 NPX (1.101) is significantly higher (*P* < 4 × 10^–37^) than those who report other ethnicities (weighted mean NPX 0.090) (Fig. 1, Table 2). About half of this difference can be attributed to the overrepresentation of G1 and G2 alleles in this cohort, which are rare or absent in non-sub-Saharan African populations. However, among Black/Black British participants with the G0/G0 genotype, mean APOL1 NPX (0.60) was higher than 88% of the UK Biobank population (β = −0.50; 95% CI (−0.55, −0.44); OR 0.61; *P* = 1.1 × 10^−65^) also 73% higher (*P* < 2x10^−16^) than the remainder of UK Biobank participants with that genotype (mean = 0.07 NPX). These findings indicate that populations with recent sub-Saharan African possess additional genetic or environmental factors that increase APOL1 NPX, or that non-African populations possess factors that reduce APOL1 NPX.

In order to investigate these other factors we first controlled for any effect non-synonymous variants may have on Olink NPX measurements by affecting antibody binding rather than true circulating APOL1 abundance. To do this, we restricted the analysis to participants who were homozygous for G0 (at both G1 and G2 loci) and the A allele at E150K and compared mean APOL1 NPX between ethnic groups (Table 2). In this subset, differences in NPX are less likely to reflect structural effects on antibody recognition and are therefore more likely to reflect underlying biological differences affecting APOL1 NPX arising from regulatory or other non-coding variation. In Black/Black British participants, the mean APOL1 NPX was 0.349 in this subset, compared with 1.10 when all genotypes were included, demonstrating that coding variation accounts for a substantial proportion of the elevated APOL1 NPX observed in this population.

We also tested whether the differences in APOL1 NPX still differed between pairs of ethnicities after controlling for genotype at these three variants (Table 3). All pairwise associations remained significant (except Asian: Chinese contrast) but were not as strong due to smaller sample and effect sizes. The remaining significant associations indicate that additional factors, including non-coding regulatory variation, also contribute to differences in plasma APOL1 NPX between populations.

### Evidence that N264K is detected in G0 and G2 haplotypes, but not G1 haplotypes

UK Biobank participants with self-reported sub-Saharan African ancestry includes 6862 individuals with an identifiable genotype at the N264 locus. It had previously been reported that the N264K variant occurs on G0 and G2 haplotypes as a result of two independent mutational events, and that N264K is mutually exclusive with the *APOL1* G1 allele [11]. Our analysis of the UK Biobank data supports this observation: N264K was observed on a G0/G0 background in 77/2639 individuals (2.9%), and on a G2/G2 background in 7/129 individuals (5.4%). N264K was not observed on a G1/G1 background 0/598 individuals (0.0%) (Table 5). Among individuals with the G0/G0 genotype, the presence of N264K had no effect on APOL1 NPX (*P* = 0.89).

**Table 5:** Distribution of N264K haplotypes across *APOL1* genotypes.

Genotype	Number in cohort	0 N264K haplotypes (%)	1 N264K haplotype (%)	2 N264K haplotypes
G0/G0	2639	2562 (97.1)	77 (2.9)	0 (0.0)
G0/G1	2069	2026 (97.9)	43 (2.1)	0 (0.0)
G0/G2	1124	1050 (93.4)	72 (6.4)	2 (0.2)
G1/G1	598	598 (100)	0 (0.0)	0 (0.0)
G1/G2	303	285 (94.1)	18 (5.9)	0 (0.0)
G2/G2	129	122 (94.6)	7 (5.4)	0 (0.0)

We examined whether N264K modified APOL1 NPX on different G1 and G2 haplotype backgrounds using a linear model adjusted for age, sex and genetic principal components. N264K was not significantly associated with APOL1 NPX on any G1 or G2 haplotype background (Table 6). Consistent with this, adding N264K to a model containing G1 and G2 haplotypes, age, sex and genetic principal components did not improve model fit (likelihood ratio test (ANOVA *P* = 0.82)) and produced virtually no change in the proportion of variance explained (R^2^ = 0.5755 versus 0.5766).

**Table 6:** Effect of the N264K variant on APOL1 NPX in different APOL1 genotype backgrounds. Mean APOL1 NPX is shown for participants without and with at least on N264K allele. Beta coefficients represent the effect of N264K within each genotype background. NPX values are reported on a log2 scale. No participants of African descent carrying Olink data were homozygous for N264K A allele: All N264K carriers were heterozygotes.

*APOL1* Genotype	Participants with N264K	Mean APOL1 NPX (0 N264K alleles)	Mean APOL1 NPX (≥1 N264K alleles)	Beta (95% CI)	*P* value
G0/G0	16	0.61	0.67	0.02 (−0.28, 0.32)	0.89
>0 G1 alleles (G0/G1, G1/G1, G1/G2)	9	1.23	1.20	−0.02 (−0.30, 0.33)	0.91
1 G2 allele (G0/G2, G1/G2)	6	2.25	2.30	0.03(−0.21, 0.27)	0.81

### Loci that regulate APOL1 NPX in white and black British populations

In the White British and Irish populations, eleven distinct protein quantitative trait Loci (pQTL) have been associated with APOL1 concentration [16] (Table S2). However, only three pQTL were identified in the Black/Black British population (Table S3). We reanalysed these associations to determine whether they might contribute to the difference between participants with recent sub-Saharan African ancestry and individuals of other ethnicities. At the two loci of largest effect in the White British/Irish population (Chr16, rs763665, OR 1.7; Chr22, rs2239785, OR 1.4), the allele associated with higher NPX was more common in Black/Black British than White British/Irish participants (Table S2). These alleles would therefore be expected to contribute to the higher APOL1 NPX in the Black/Black British group. Despite this prediction, no association was detected in the GWAS for APOL1 NPX in that population (Table S3). The effects of the remaining nine loci were very small: some increased and some reduced APOL1 NPX in the Black/Black British population relative to the White British/Irish population (Table S2).

The strongest associations with APOL1 NPX were in the region of APOL1 but only one SNP was reported to be associated with APOL1 NPX in each population [16]. Additional *cis*-acting loci might be masked by the signal from the lead SNP in each population [14]. We therefore ran repeated association analyses for each population, after each iteration we added the variant with the smallest p-value to a list of conditioning SNPs for the next iteration until the smallest p value was > 10^−7^. There were four independently associated *cis-*SNPs linked to APOL1 NPX in the White British population and three in the Black/Black British population (Table 7, Table S4, Fig. 2).

**Table 7:** Independent cis associations with APOL1 NPX identified by iterative conditional association analysis. Iteration 1 included sex, age, and the first 4 genetic principal components as covariates. At each subsequent iteration, the lead SNP from the previous analysis included as an additional covariate until no SNP remained associated at *P* < 1 × 10^−7^.

Iteration	Position (bp)	Lead SNP	Linkage Group	MAF	Beta	Beta 95% CI	*P*
**White British**							
1	36,660,842	rs136168	9	0.19	0.17	0.16, 0.18	1.4 × 10^−200^
2	36,649,966	rs6000220	6	0.11	−0.10	−0.12, −0.09	8.1 × 10^−50^
3	36,657,789	rs136164	10	0.40	−0.04	−0.05, −0.02	1.1 × 10^−10^
4	36,639,067	rs13054261	3	0.19	−0.04	−0.06, −0.03	5.6 × 10^−06^
**Black/Black British**							
1	36,662,041	rs71785313 (G2)	4	0.11	1.13	1.06, 1.2	1.3 × 10^−145^
2	36,661,906	rs73885319 (G1)	-	0.24	0.33	0.27, 0.38	8.6 × 10^−30^
3	36,661,330	rs2239785 (E150K)	3	0.35	−0.15	−0.2, −0.09	5.4 × 10^−07^

**Figure 2:**
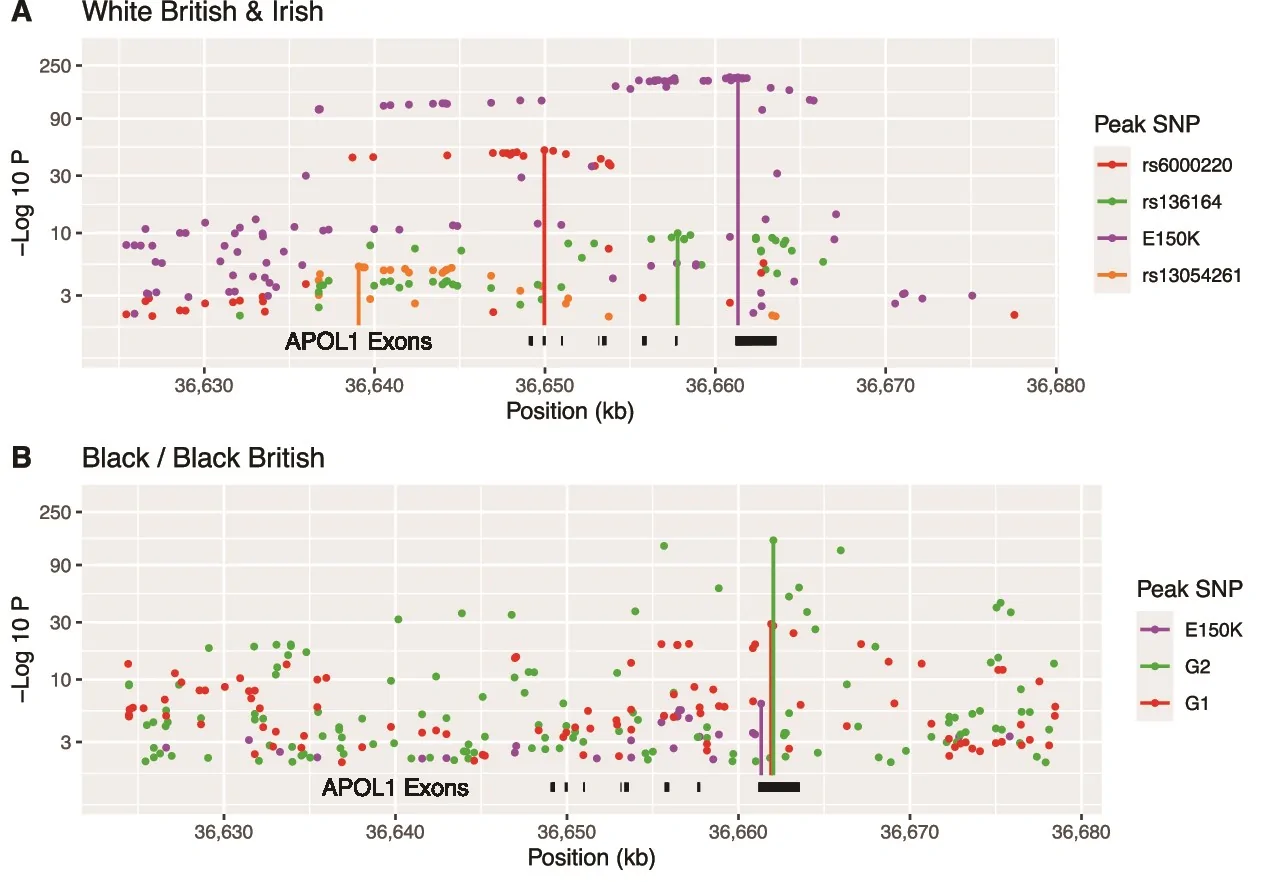
Regional association plots showing SNP associations with APOL1 NPX in white British/Irish and black/black British participants. Results were obtained by iterative conditional association analysis. At each iteration, the SNP with the smallest *P* value from the previous analysis was added to a list of conditioning SNPs before repeating the association analysis. The y-axis shows -log10 p values for association. A log–log scale was used to compress the strongest associations and allow both strong and weak association signals to be visualised on the same plot. Colours indicate SNPs associated with each successive lead SNP after controlling for more strongly associated variants. Vertical lines indicate the lead SNP identified at each iteration. In the black/black British population the only variants independently associated with APOL1 NPX were missense variants in the final exon, with E150K showing the smallest effect. In the white British/Irish population, E150K was the only independently associated missense variant. For clarity, rs2239785 (E150K) is shown in place of rs136168 (Table S1), which had the smallest p value but is in very strong linkage disequilibrium with rs2239785, and likely represents the same underlying functional signal.

The top two SNPs in the White British population have previously been associated with APOL1 NPX or are in the same linkage group as SNPs previously associated with APOL1 NPX (Table S4), the other two SNPs (rs136164 and rs13054261) have small and negative βs (−0.04) but high minor allele frequencies (Table 7) and could contribute to the lower APOL1 NPX of the White British population.

The top two SNPs in the Black/Black British population were G1 and G2, the third, E150K (rs2239785) was a common SNP (MAF 35%) with a relatively large negative β (−0.15) that is predicted to reduce APOL1 NPX in this population.

Interferon gamma (IFNG) has been shown to stimulate APOL1 expression [17], but in a linear regression model with sex, age, and the first four genetic principal components as covariates there was no evidence for IFNG NPX being associated with APOL1 NPX in the Olink data (*P* = 0.18).

### New SNPs associated with APOL1 NPX

The published list of SNPs associated with Olink APOL1 NPX only includes three *cis*-acting SNPs in the *APOL1* region; rs2239785 (E150K) and rs6000220 in the White British/Irish population, and rs71785313 (G2) in the Black/Black British populations respectively [14, 16]. In order to identify additional SNPs that might independently regulate APOL1 NPX we undertook an association study of all SNPs within 500 kb of the start and end of *APOL1*. 164 SNPs were associated with APOL1 NPX in the White British/Irish population with *P* < 5x10^−8^ (Table S5). Nineteen of these SNPs in 5 linkage groups were still associated after conditioning on rs2239785 and rs6000220 which are the only known SNP associated with APOL1 NPX in the White British/Irish population (Table S6). The associations between these five linkage groups and APOL1 NPX are therefore most likely to be due to regulatory SNP. Ninety-five SNPs were associated with APOL1 NPX in the Black/Black British population (Table S7) but conditioning on G2, G1 and rs2239785 (E150K) left no further significant associations.

### SNPs in microRNA binding sites may reduce APOL1 NPX in the white British population

In order to test whether the putative regulatory variation might be mediated by variants in microRNA binding sites, a list of microRNA binding sites in the 3′ untranslated regions of *APOL1* was downloaded from mirDB [18], and each binding site was tested for the presence of SNP loci with significant associations with APOL1 NPX (*P* < 5 × 10^−8^). Associations with each SNP in each predicted microRNA binding site are shown in Table S5, with the corresponding predicted microRNAs shown in Table S8, and a summary is shown in Table 8. Six SNPs within a single linkage group were associated with slightly lower (mean β = −0.05) APOL1 NPX in the White British population. Predicted functional effects of each SNP are shown in Table S9. None of the SNPs in the relevant linkage group was a non-synonymous SNP that might modify Olink probe binding and these SNPs remained associated with APOL1 NPX after conditioning on rs2239785 (E150K) (Table S6) which effectively controls for all the non-synonymous SNPs known to be associated with APOL1 NPX. There were no SNPs in the Black British population that were in microRNA binding sites and robustly associated with APOL1 NPX after controlling for linkage to APOL1 non-synonymous SNPs. These findings suggest that variation in miRNA binding sites may contribute to population differences in APOL1 NPX but do not explain the large differences observed between Black and White British populations.

**Table 8:** SNPs within predicted microRNA binding sites in the *APOL1* 3′ untranslated region that remain associated with APOL1 NPX in the white British population after controlling for the effect of missense SNPs linked to rs2239785 (E150K). ‘Predicted miRNA binding sites’ indicates the number of predicted microRNA binding sites that overlap each SNP. See Table S8 for the corresponding predicted microRNAs and Table S6 for the complete list of SNPs associated with APOL1 NPX after controlling for linkage to missense SNPs.

ID	Position in APOL1 3’ UTR (bp)	Predicted miRNA binding sites (n)	MAF	Beta	Beta 95% CI	*P*
rs9610472	292	3	0.14	−0.04	−0.051, −0.026	7.4 × 10^−10^
rs9610473	298	3	0.14	−0.04	−0.052, −0.027	4.0 × 10^−10^
rs9610474	307	11	0.14	−0.04	−0.051, −0.026	7.9 × 10^−10^
rs9610475	598	1	0.17	−0.03	−0.044, −0.021	1.2 × 10^−08^
rs9610476	600	1	0.17	−0.03	−0.044, −0.021	1.7 × 10^−08^
rs1142542	1254	4	0.17	−0.04	−0.048, −0.025	9.9 × 10^−11^

## Discussion

Recent laboratory and association studies have transformed our understanding of the human *APOL1* gene. They have widened the scope of potentially detrimental effects in current populations as an evolutionary cost of surviving historic sleeping sickness epidemics [7, 12, 19–21], and have identified graded and differential risk associated with specific alleles, allele number, and modifier variants that mitigate APOL1 impact both on trypanosomiasis and CKD [8–11]. Here, we further extend those findings to demonstrate that *APOL1* G1 and G2 are both associated with dose-dependent increases in APOL1 NPX, however the effect of G2 is far larger (Table 4).

Recent analyses have demonstrated that *APOL1* coding variants can generate platform-dependent effects in plasma proteomic assays, with discordant directions of association observed across affinity-based platforms and no significant effect detected using mass spectrometry [14]. These findings suggest that affinity-based measurements may reflect conformational or epitope-specific properties of APOL1 rather than absolute circulating protein abundance. A previous version of the present study was made publicly available on MedRxiv prior to publication of that report and similarly described genotype-dependent differences in APOL1 NPX [22]. Our analyses therefore characterise the genetic architecture of APOL1 NPX signal and do not assume that the signal represents total circulating APOL1 protein. As NPX represents a relative affinity-based signal, our findings should not be interpreted as direct measures of absolute circulating APOL1 concentration.

Wang et al. [14] showed that the non-synonymous variants G1, G2 and rs2239785 (E150K) were strongly but inconsistently associated with APOL1 NPX in the Olink and Somalogic affinity assays but not associated with APOL1 abundance in mass spectrometry-based assays. Although there are other non-synonymous polymorphisms in *APOL1,* we found no associations between any of these and APOL1 NPX in either the White or Black British populations that were not accounted for by linkage to G2, G1 or rs2239785 (E150K). Despite these caveats, the higher APOL1 NPX observed in Black/Black British participants who are homozygous for the reference allele at G1, G2, and rs2239785 (E150K) than in White British participants with the same genotype cannot be attributed to coding variant-induced conformational effects. We followed up on the work of Wang et al. [14] with two strategies to identify all potential independent cis regulators of APOL1 NPX. First, we performed iterative association analysis, conditioning each iteration on the associations identified in previous iterations. Second, we identified linkage groups within the APOL1 region and evaluated associations within each of them. This process effectively conditioned on the effects of non-synonymous SNPs that appear to modify APOL1 NPX without necessarily altering APOL1 protein abundance. After controlling for the non-synonymous SNPs, the difference in APOL1 NPX between White and Black British populations declined from 1.03 to 0.34 (Table 2). The remaining difference in APOL1 NPX between these populations was still highly significant (*P* = 2 × 10^−8^) (Table 3) and may reflect regulatory variation and other genetic or environmental factors. In addition, the identification of multiple non-coding cis-regulatory variants associated with APOL1 NPX indicates that the observed variation in APOL1 NPX is not just attributable to assay artefacts.

However, even if affinity-based measurements do not directly reflect absolute circulating APOL1 concentration, the consistent genotype-specific differences observed here remain biologically informative. The G1/G1, G1/G2, and G2/G2 genotypes produce clearly distinct APOL1 NPX distributions. If these differences arise from altered antibody binding rather than absolute abundance, they still imply that APOL1 protein variants encoded by these genotypes differ in structural or biochemical properties. Such differences are themselves physiologically meaningful and provide additional evidence that G1 and G2 variants, and the genotypes they generate, are not functionally equivalent.

These observations are consistent with findings from infection biology and human genetics which indicate that G1 and G2 have distinct functional consequences. In studies examining the role of APOL1 in trypanosomiasis, the different effects of the two variants are well-established: G1 is associated with protection against severe disease in *T.b. gambiense*; G2 is associated with protection from *T.b. rhodesiense* infection, but is associated with increased disease severity in *T.b. gambiense* [3]. Accordingly, trypanosomiasis studies routinely consider G1 and G2 separately. In contrast, CKD studies have typically grouped G1/G1, G1/G2, and G2/G2 together as ‘high-risk’. Recently, we demonstrated that this approach masks a deeper level of complexity, and identified conditions and phenotypes within CKD and beyond that were associated with particular two-variant *APOL1* genotypes [7]. Here we extend these findings by demonstrating genotype-specific associations with APOL1 NPX levels, and their modifiers.

The role of *APOL1* G2 in trypanosome infections is well documented. In *T.b. rhodesiense*, the parasite serum resistance-associated (SRA) protein binds wild-type APOL1 and blocks APOL1-mediated trypanolysis. However the two amino acid deletion present in the G2 variant modifies APOL1 to prevent SRA binding, restoring APOL1’s lytic function and conferring protection [23]. Similarly, G2-specific phenotypes have been observed in CKD, with the G2/G2 genotype being associated with reduced (<60 ml/min/1·73m^2^) estimated glomerular filtration rate [7]. The mechanisms resulting in APOL1-mediated cell injury in CKD are unclear: multiple pathways have been proposed, and studies have typically examined genotypes G1/G1, G1/G2, and G2/G2 collectively as ‘high-risk’. If the causal mechanisms of CKD differ between G1 and G2, treating the variants as equivalent in studies will hinder efforts to further understand the pathogenesis of CKD.

Phenome-wide analyses also reinforce this point. We previously found the G1/G2 genotype to be associated with multiple deleterious conditions (as defined by *International Classification of Disease* (ICD) codes) [7], while detecting no association with G2/G2 (either protective or deleterious). This was unexpected given that in mouse BAC transgenic models with the human promoter regions, G2/G2 has been shown to be associated with more severe kidney phenotypes and that this effect is dose-dependent [24]. The absence of associations with G2/G2 in our phenome scan could reflect reduced statistical power given the relative rarity of G2/G2. However, the effect size for G2/G2 was smaller than that of the index genotype with a significant association in all cases and below the 95% confidence interval of the index association in 19 of 27 cases in our previous phenome-wide screen (Table S10) [7]. This indicates that lack of power for the rarer G2/G2 genotype is unlikely to explain the absence of associations with this genotype. Therefore, either the G2/G2 genotype is not a risk factor for many conditions mediated by mechanisms other than CKD, or the effects of G2 homozygosity are masked by such factors as tissue-specific concentration, altered substrate binding, protein folding variation or other biochemical properties that drive disease risk.

The N264K variant further illustrates the complexity of APOL1 biology. In a G2 background, N264K reduces trypanolytic function [8] and appears to protect against G2-associated kidney disease by disrupting APOL1 G2 pore-forming and ion channel conduction [10, 11]. In our analysis of UK Biobank genotype data, the N264K variant had no effect on APOL1 NPX on any genotype background, including backgroups containing G2. By contrast. Wang et al. [14] observed a reduction in APOL1 NPX associated with N264K and G2 in combined UK Biobank exome and genotype data. Their association was driven by participants homozygous for both G2 and the N264K A allele. We identified no such individuals in the subset of genotype and Olink data analysed here, suggesting that larger datasets will be required to evaluate this genotype combination reliably.

The data presented here also highlight additional contributors to APOL1 expression. Even after controlling for known non-synonymous variants, those with sub-Saharan African ancestry had significantly higher APOL1 NPX than individuals with other ethnicities (Table 2). Therefore, the conformational effects described in recent cross-platform studies cannot explain this residual difference in APOL1 NPX [14]. This indicates that APOL1 NPX is influenced by additional factors. It has already been shown that there are 10 trans-acting pQTLs regulating APOL1 NPX in the White British population but only 2 trans pQTLs in the Black/Black British population (Tables S2 and S3) [16]. At the two loci of largest effect, the major allele was associated with reduced APOL1 NPX in the White British population. A separate study of *cis*-regulation of APOL1 NPX in the White British population [14] reported two SNPs associated with APOL1 NPX, rs2239785 (E150K) was associated with higher APOL1 NPX and rs6000220 was associated with lower APOL1 NPX. We conditioned our association analysis on these two SNPs and found associations (*P* < 5 × 10^−8^) in four different linkage groups (Table S6). Although the effect size of each linkage group was small (β = ~ −0.04) they were all associated with lower APOL1 NPX. The combined effect of the published SNPs [14] and the additional loci we identify here may account for much of the difference in APOL1 NPX between populations but functional studies will be required to determine whether these candidate variants directly regulate APOL1 and to establish the direction of their effects.

MicroRNAs suppress expression of their target proteins, and we identify variants within predicted microRNA binding sites that may contribute to differences in APOL1 NPX. The residual population difference in APOL1 NPX might also be attributable to many loci of small effect as well as environmental factors that were not incorporated into our model. The identification of additional loci and environmental factors that influence APOL1 NPX, and an assessment of their impact on human health would further develop our understanding of the protein and inform diagnostics and management for APOL1-related conditions.

There are some limitations to the study. The analysis is cross-sectional, and we therefore cannot determine the longitudinal independent association with CKD, and circulating APOL1 NPX does not necessarily reflect tissue levels of APOL1 that could be more important for kidney damage. In addition, our analyses rely on Olink-based measurements and therefore describe Olink-measured APOL1 NPX signal rather than absolute circulating protein abundance. Independent validation using complementary proteomic approaches would further clarify the relationship between NPX signal and total APOL1 concentration. Our main strength is the large, well categorised study population, which enabled us to quantify population- and genotype-specific differences in APOL1 NPX with precision.

## Conclusions

This study demonstrates the relationship between *APOL1* variants and circulating APOL1 NPX. It identifies further phenotypic differences between the G1 and G2 variants, reinforcing the importance in considering them as biologically distinct in molecular and association studies. In addition, we define regulatory and modifier effects that shape circulating APOL1 NPX. Together, these findings indicate that *APOL1* genotype contributes to a structured circulating APOL1 signal shaped by both coding and regulatory variation. Understanding this genetic architecture provides a framework for interpreting the molecular and regulatory consequences of APOL1 variation and may inform future strategies for risk stratification and therapeutic development in APOL1-associated disease.

## Materials and methods

### Ethical approval

Access to the UK Biobank was granted for this work under UK Biobank application number 66821.

### Study design and participants

The UK Biobank is a prospective cohort study of 502 460 adults aged 40 to 69 years at enrolment between 2006 and 2010 from 22 assessment centres across the United Kingdom [25]. At the baseline study visit, participants underwent nurse-led interviews and completed detailed questionnaires about medical history, medication use, sociodemographic factors, and lifestyle in addition to a range of physical assessments and provided blood and urine. The UK Biobank study was approved by the North-West Multi Centre Research Ethics Committee, and all participants provided written informed consent.

### UK biobank self-reported ethnicity data

Self-reported ethnicity data was obtained from UK Biobank data field 21 000. Descriptors of ethnicity used here (Asian or Asian British, Black or Black British, Chinese, Mixed, and White) represent the top-level descriptors of ethnicity used in data field 21 000.

### UK biobank Olink protein quantification data

Olink protein quantification was performed on 53 018 UK Biobank participants [16]. APOL1 protein quantification data is available for 44 840 of these. Participants with missing or anomalous haplotypes at the G1 and G2 loci (such as those who were homozygous for one and heterozygous for the other) were excluded as were those without a specifically defined ethnicity. After applying these filters 43 587 participants were included in the analysis. Olink data is expressed in the Normalized Protein eXpression (NPX) scale, enabling relative quantification of the same protein across multiple samples. As NPX values are calculated on a log2 scale, an NPX difference of 1 represents a doubling of protein concentration. Throughout this manuscript, ‘APOL1 NPX’ refers to the normalised protein expression value obtained from the Olink platform and represents a relative measure of circulating APOL1 signal rather than an absolute concentration.

### Genotyping

*APOL1* genotypes were obtained from the UK Biobank, which used a custom Affymetrix array for the G1 (rs73885319) and N264K (rs73885316) alleles. G2 (rs71785313) genotypes were imputed by the UK Biobank as previously described [25]. The study includes only participants with complete, unambiguous *APOL1* G1 and G2 genotype data.

### Statistical analysis

APOL1 NPX was tested for association with *APOL1* genotypes with, age, sex, and genetic principal components 1–4 as covariates. Linear regression as implemented in R was used to compare the effects of *APOL1* genotypes and individual risk alleles. All statistical tests were 2-sided, and *P* < 0.05 was considered statistically significant.

### Identification of regulatory SNPs associated with APOL1 NPX

The published study of SNPs associated with APOL1 NPX only reports a single SNP within each QTL [16]. To identify additional SNPs that might regulate APOL1 NPX we tested for associations between 21 406 SNPs and APOL1 NPX. SNPs were from the UK Biobank Affymetrix genotype data and within 500 Kb upstream and downstream of *APOL1*. Association testing was performed using Plink2 [26] with a general linear model [27], using age, sex, and the first four principal components of the relevant sample set as covariates. SNPs that were associated with APOL1 NPX (*P* < 5 × 10^−8^) were tested for linkage with each other. Linked SNPs were clustered into groups where each locus was linked to at least one other in the group using the NetworkX package in Python [28].

To understand the mechanism of APOL1 NPX regulation, we tested whether

the SNPs that were associated with APOL1 NPX in the Black/Black British and White populations were also within microRNA binding sites. A list of microRNA binding sites was downloaded from mirDB [18], and each binding site was tested for the presence of SNP loci with significant associations with APOL1 NPX using a custom local Perl script.

## Supplementary Material

Supplementary_Data_August_2026_ddag087

## Data Availability

This research has been conducted using data from the UK Biobank, a major biomedical database: www.ukbiobank.ac.uk.
